# Tissue culture and rapid propagation technology for *Gentiana rhodantha*

**DOI:** 10.1515/biol-2022-0565

**Published:** 2023-05-18

**Authors:** Suju Han, Mei Liu, Yuetong Wang, Juan Chen

**Affiliations:** Engineering Research Center of Chuanxibei RHS Construction at Mianyang Teachers’ College of Sichuan Province, School of Urban and Rural Planning and Construction, Mianyang Teachers’ College, Mianyang, 621000, China; Ecological Security and Protection Key Laboratory of Sichuan Province, Mianyang Teachers’ College, Mianyang, 621000, China

**Keywords:** *Gentiana rhodantha*, rapid propagation, tissue culture

## Abstract

*Gentiana rhodantha* is a perennial herb of the genus *Gentiana* (Tourn.) L. This study was novel in establishing a regeneration system of *G. rhodantha* using young leaves as explants on the Murashige and Skoog (MS) medium supplemented with different plant growth regulators (PGRs). The roots, stems, and leaves of *G. rhodantha* were used as explants. The effects of the optimal explant disinfection method, type of explant used, concentrations of PGRs added to the culture media on tissue culture, and rapid propagation of *G. rhodantha* were studied. The results showed that the optimal disinfection method for stems and roots consisted of disinfection using 75% ethanol for 50 s, followed by 4% sodium hypochlorite (NaClO) for 10 min. The optimal disinfection technique for leaves consisted of disinfection using 75% ethanol for 50 s, followed by 4% NaClO for 8 min. Root explant was the most suitable for inducing the callus of *G. rhodantha* on the MS medium supplemented with different PGRs. The optimal conditions for callus induction included 1.0 mg/L 6-benzylaminopurine (6-BA) and 0.5 mg/L α-naphthalene acetic acid (NAA). The callus induction rate using the root explant reached 94.28%. MS supplemented with 2.0 mg/L 6-BA and 0.1 mg/L NAA was the optimal medium for inducing adventitious shoots from the callus of *G. rhodantha*. The best medium for propagation and plantlets strengthening was MS supplemented with 0.8 mg/L 6-BA and 0.3 mg/L NAA, and the propagation index was 8.62. MS supplemented with 0.3 mg/L 3-indolebutyric acid was the best culture medium for inducing the rooting of adventitious buds, with the maximum rooting rate reaching up to 100%.

## 1 Introduction

*G*entiana *rhodantha* Franch. is a perennial herb of the genus *Gentiana* (Tourn.) L. and a newly catalogued medicinal herb in the 2015 Chinese Pharmacopoeia [1]. *G. rhodantha* is an endemic plant in China and rich in compounds such as mangiferin, flavonoids, and sterols. Mangiferin is known for its definite efficacy in relieving cough and inflammation. In recent years, the demand for *G. rhodantha* as a raw material has grown. It is part of the folk tradition to use *G. rhodantha* to treat pneumonia, bronchitis, and hepatitis [2,3]. *G. rhodantha* plantlets are short and have many branches. This plant blooms in winter, and the flowers are bright-colored and last long. It can also be used for urban landscaping in winter or grown in pots for indoor decoration [4]. However, seed germination can be tricky in the case of *G. rhodantha*. Given its reproductive capacity, *G. rhodantha* is commercially propagated by cutting its top buds. However, this propagation method is usually associated with slow rooting and poor growth. Sexual reproduction, which involves a long cycle, can be considerably restricted by the natural environment. Besides, the reproduction rate and the plant quality are low with split cultivation, making *G. rhodantha* unsuitable for mass production [5]. The conventional propagation techniques for *G. rhodantha* are time-consuming, involve complex procedures, and have low yields, and therefore can hardly meet the market demand. On the contrary, the propagation system of *G. rhodantha* is suitable for mass production to meet the increasing market demand [6].

The recent studies on *G. rhodantha* mainly focus on the following aspects: germplasm resource investigation [7], chemical composition analysis [8,9], pharmacological research [3–12], and localization of suitable areas [13]. On the contrary, few studies involve tissue culture of *G. rhodantha*. Zhong built a rapid propagation system of *G. rhodantha* using nodal segments [14]. The callus induction and adventitious bud differentiation for *G. rhodantha* are much less discussed. Tissue culture is featured by the high propagation index and controllability of culture conditions. These two advantages can greatly accelerate the propagation of *G. rhodantha*, contributing to the popularization of this species.

In this study, the young and tender roots, stems, and leaves of *G. rhodantha* were used as explants. The callus induction, adventitious shoots differentiation, and rooting of this plant species were examined using the optimal disinfection method and the optimal hormone ratio. We replaced the conventional ethanol-corrosive sublimate combination for disinfection with sodium hypochlorite (NaClO) to reduce environmental pollution. On this basis, we built the rapid propagation system of *G. rhodantha*, which laid the foundation for the rapid propagation of this species, alleviated the shortage of wild *G. rhodantha* resources, and created favorable conditions for developing ornamental *G. rhodantha.*

## 2 Materials and methods

### 2.1 Experimental materials

The plants of *G. rhodantha* were brought from Daba Village, Huize County, Qujing City of Yunnan Province. They were transported to the Mianyang Normal University of Sichuan Province, People’s Republic of China (104°78′E, 31°5′N). All culture media were supplemented with 30 g/L sucrose and 7.5 g/L agar. All media were adjusted to pH 5.8 with 0.1 N sodium hydroxide solution before autoclaving at 121°C for 20 min. All explants and plantlets were cultured at 25 ± 2°C under 16 h photoperiod and 2,000 lx light intensity.

### 2.2 Experimental method

#### 2.2.1 Explant disinfection

The roots, young stems, and young leaves of *G. rhodantha* were used as explants. The explants were washed with running water for 1–2 h and then rinsed with sterile distilled water for 3 min.

On an ultraclean workbench, the stem, root, and leaf explants were decontaminated under aseptic conditions using 75% ethanol for 40, 50, and 60 s, respectively. Then, the explants were dipped in 4% NaClO solution and shaken for 8, 10, and 12 min, respectively. Next, the explants were washed using sterilized water five to six times and cut into 1 cm-long segments. They were inoculated into the Murashige and Skoog (MS) medium supplemented with 0.5 mg/L 6-benzylaminopurine (6-BA) and 0.5 mg/L α-naphthalene acetic acid (NAA). The orthogonal experimental design was adopted, with nine different treatments established. One explant was inoculated into each bottle. Ten explants were inoculated for each treatment, thus totaling 90. After 1 month, contaminated explants for each treatment were identified and their optimal disinfection duration was determined. The optimal disinfection was used in the subsequent experiments.

#### 2.2.2 Screening for the optimal culture medium for callus induction

The MS medium was supplemented with different concentrations of 6-BA (at the concentration of 0.5, 1.0, and 1.5 mg/L, respectively) and NAA (at the concentration of 0.5, 1.0, and 1.5 mg/L, respectively) for the stem, root, and leaf explants. Nine treatments were set up using the orthogonal design. After disinfection, three sterile explants were inoculated into one culture bottle. Each treatment encompassed three bottles, with three replicates. Thus, 81 bottles were used in total. The growth status was recorded on Day 30. The explants most suitable for callus induction and the optimal hormone concentration combination for this purpose were identified.

#### 2.2.3 Screening for the optimal culture medium inducing adventitious shoot regeneration

The vigorously growing calluses were inoculated to the MS media supplemented with different concentrations of 6-BA (at a concentration of 1.5, 2.0, and 2.5 mg/L, respectively) and NAA (at a concentration of 0.1, 0.2, and 0.3 mg/L, respectively) for the stem, root, and leaf explants. Nine treatments were set up using the orthogonal design. The explants were inoculated into three bottles for each treatment (under the same culture conditions as earlier), with three replicates. Thus, 81 bottles were used in total. The growth of adventitious buds was observed, and the induction rate was estimated 3 weeks later. The optimal culture medium for inducing adventitious bud differentiation from the callus of *G. rhodantha* was identified.

#### 2.2.4 Screening for the optimal culture medium for the proliferation of adventitious shoots

The vigorously growing adventitious shoots were inoculated into the MS media supplemented with different concentrations of 6-BA (at a concentration of 0.8 and 1.2 mg/L, respectively) and NAA (at a concentration of 0.1, 0.3, and 0.5 mg/L, respectively). Six combinations were set up using the orthogonal design. The explants were inoculated into three bottles for each treatment, with three replicates. Thus, 54 bottles were used in total. After 1 month, the growth status was observed, and the propagation index was calculated.

#### 2.2.5 Screening for the optimal culture medium for rooting

The vigorously growing adventitious shoots were inoculated to the MS media supplemented with IBA at a concentration of 0.1, 0.3, and 0.5 mg/L, respectively. Thus, three treatments were set up. The shoots were inoculated to three bottles for each treatment (under the same culture conditions as earlier), with three replicates. Thus, 27 bottles were used in total. The root growth was observed for 20 days, and the growth status was evaluated and subjected to statistical analysis.

#### 2.2.6 Data processing

The data were statistically processed using Excel 2007 and SPSS 17.0. The experimental results were subjected to analysis of variance.

1. Contamination rate = Number of contaminated explants/number of inoculated explants × 100%
2. Browning rate = Number of browning explants/number of inoculated explants × 100%
3. Death rate = Number of dead explants/number of inoculated explants × 100%
4. Survival rate = Number of surviving explants/number of inoculated explants × 100%
5. Callus rate = Number of explants from which the callus was induced/number of surviving inoculated explants under aseptic conditions × 100%
6. Adventitious shoots differentiation rate = Number of calluses from which adventitious shoots were differentiated/total number of inoculated calluses × 100%
7. Propagation index = Number of shoots induced by propagation/number of inoculated shoots × 100%

## 3 Results and analysis

### 3.1 Effects of disinfection duration on the explants

As shown in Table 1, the duration of disinfection with ethanol and NaClO was closely related to the explant contamination rate, browning rate, and death rate. Specifically, the longer the duration of disinfection with ethanol and NaClO, the lower the contamination rate and higher the death and browning rates. Taken together, the optimal disinfection method for the stem and root segments consisted of disinfection with 75% ethanol for 50 s, followed by 4% NaClO for 10 min. For leaves, the optimal disinfection method consisted of disinfection with 75% ethanol for 50 s, followed by 4% NaClO for 8 min.

**Table 1:** Effects of different disinfection methods on the explants of *G. rhodantha*

Treatment serial no.	Explant	75% Ethanol (s)	4% NaClO (min)	Number of bottles in which explants were inoculated	Number of dead explants	Number of contaminated explants	Number of browned explants
1	Leaves	40	8	20	14	17	17
2	Leaves	40	10	20	16	15	19
3	Leaves	40	12	20	18	10	19
4	Leaves	50	8	20	15	10	12
5	Leaves	50	10	20	16	10	15
6	Leaves	50	12	20	19	9	17
7	Leaves	60	8	20	17	10	16
8	Leaves	60	10	20	19	8	18
9	Leaves	60	12	20	20	6	20
10	Stem	40	8	20	15	15	7
11	Stem	40	10	20	13	13	8
12	Stem	40	12	20	10	10	12
13	Stem	50	8	20	10	9	10
14	Stem	50	10	20	7	7	12
15	Stem	50	12	20	8	6	14
16	Stem	60	8	20	11	10	11
17	Stem	60	10	20	12	8	13
18	Stem	60	12	20	10	7	15
19	Root	40	8	20	15	15	7
20	Root	40	10	20	13	13	9
21	Root	40	12	20	10	10	10
22	Root	50	8	20	10	9	7
23	Root	50	10	20	7	7	8
24	Root	50	12	20	8	6	11
25	Root	60	8	20	11	10	10
26	Root	60	10	20	12	8	12
27	Root	60	12	20	10	5	13

### 3.2 Effects of plant growth regulators on the rapid propagation of *G. rhodantha*

#### 3.2.1 Effects of plant growth regulators on callus induction from different explants of *G. rhodantha*

**Figure 1:**
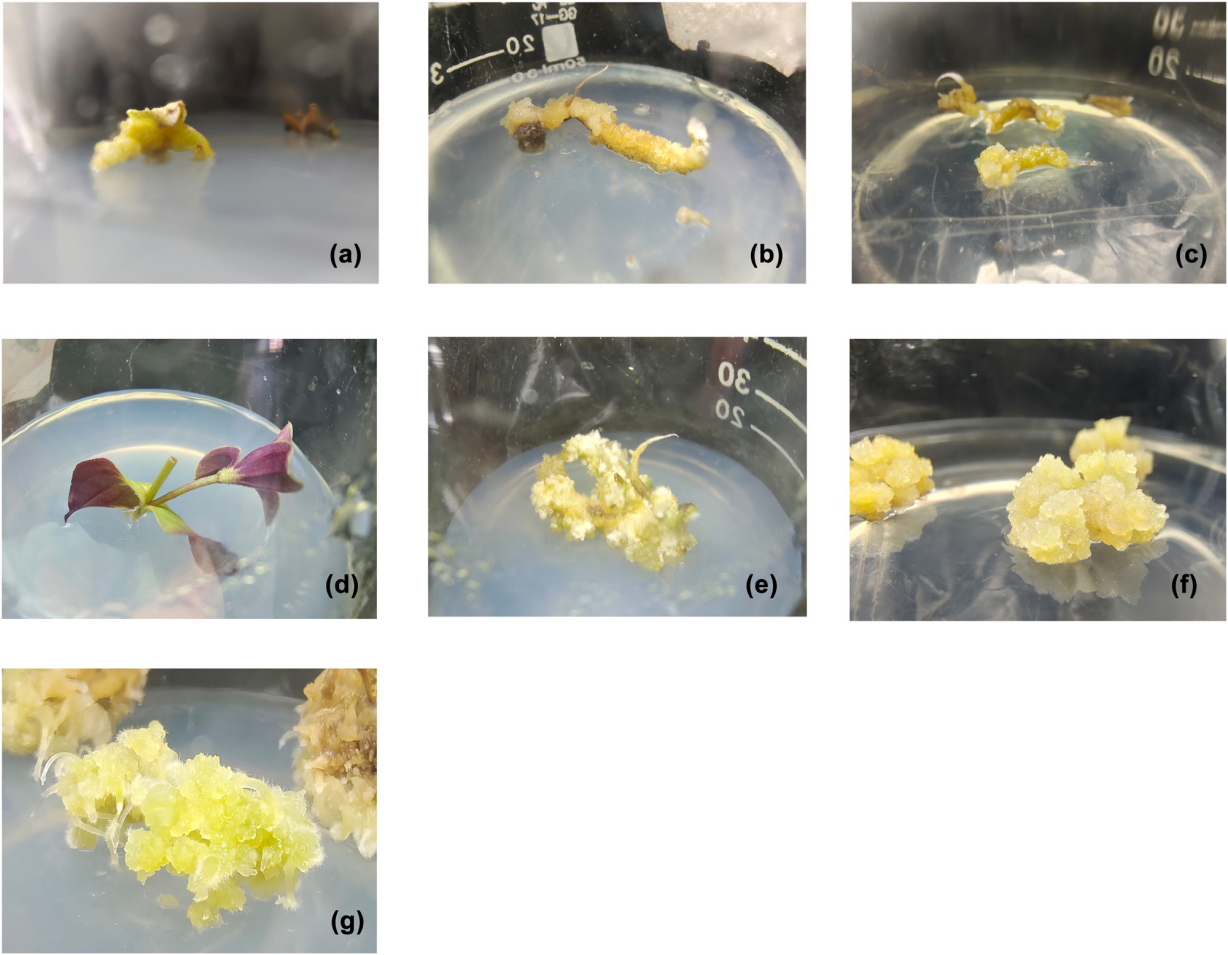
Effects of hormones on callus induction from different explants of *G. rhodantha*: (a) callus induced from leaves after 12 days, (b) yellow transparent callus induced from the root after 12 days, (c) yellow transparent callus induced from the cut stem segment after 12 days, (d) lateral bud growing out of the stem segment, (e) callus gradually covering the entire root, (f) callus induced from the root 40 days later, and (g) callus induced from the stem node growing larger.

The leaves gradually thickened and turned brittle in the last week of tissue culture. A few lateral buds began germinating in the stem segments, and the cut in the stem node gradually bulged outward. The roots turned from light yellow to green. Browning occurred in most leaves on Day 12 of tissue culture. The green color receded in the surviving leaves, and the callus grew in the cut (Figure 1a). Yellow, transparent calluses were seen at the cuts in the rhizome and some stem segments (Figure 1b and c). The lateral buds grew in the stem segments (Figure 1d). Later, the callus gradually covered the entire rhizome (Figure 1e). After 40 days of tissue culture, the rhizome was enveloped by the callus, as shown in Figure 1f. The callus of the stem node grew slowly (Figure 1g).

After 1 month, the calluses were compared between different treatments and explants. The performance of callus induction varied significantly across different combinations of hormone concentrations. Among the three types of explants, the number of browning leaves was far higher than that of browning calluses from stem nodes and roots. However, the growth rate of leaf calluses was far lower than that of calluses from stem nodes and roots. The calluses induced from different explants varied in color, density, and texture. The calluses induced from leaves were mostly yellowish white, with a hard, compact texture. The calluses induced by the stem and root segments were mostly yellowish green, with a loose texture and fast growth. As shown by the statistics in Table 2, the number of browning leaves was far higher than the number of browning calluses induced from roots and stem nodes. Therefore, yellowish-green calluses with a loose structure and fast growth could be induced by inoculating the explants to media containing 1 mg/L 6-BA and 0.5 mg/L NAA. Such calluses could be induced from roots and stem nodes with high efficiency, with the induction rate reaching 94.28%.

**Table 2:** Effects of hormones on callus induction from different explants of *G. rhodantha*

Treatment serial no.	6-BA (mg/L)	NAA (mg/L)	Leaves		Stem		Root	
			Browning rate (%)	Induction rate (%)	Browning rate (%)	Induction rate (%)	Browning rate (%)	Induction rate (%)
1	0.5	0.5	90.44 ± 5.98a	24.35 ± 6.48e	14.27 ± 2.64f	50.67 ± 4.21d	16.31 ± 2.36a	65.23 ± 1.2e
2	0.5	1.0	85.71 ± 3.64c	25.67 ± 8.27d	23.84 ± 5.98a	36.74 ± 5.69g	12.8 ± 5.66b	57.16 ± 2.36g
3	0.5	1.5	90.96 ± 4.18a	28.11 ± 4.62b	15.28 ± 5.67e	40.59 ± 4.98f	10.37 ± 4.36c	69.86 ± 5.8d
4	1.0	0.5	72.67 ± 6.98h	30.74 ± 2.64a	13.64 ± 4.16g	69.15 ± 7.21a	6.97 ± 2.96f	94.28 ± 2.33a
5	1.0	1.0	76.34 ± 7.61g	26.84 ± 5.78c	21.67 ± 5.78b	33.81 ± 6.54i	9.87 ± 6.87d	60.96 ± 6.05f
6	1.0	1.5	80.21 ± 2.36e	25.44 ± 4.98d	22.14 ± 7.56b	55.77 ± 7.49c	10.26 ± 4.26c	85.73 ± 4.55c
7	1.5	0.5	77.29 ± 3.14f	29.71 ± 3.84a	19.35 ± 7.65c	58.75 ± 4.37b	8.27 ± 7.49e	90.27 ± 5.52b
8	1.5	1.0	88.75 ± 5.27b	25.97 ± 6.24d	16.87 ± 2.79d	49.26 ± 4.81e	10.9 ± 4.79c	70.16 ± 3.56d
9	1.5	1.5	82.69 ± 8.29d	22.34 ± 4.73f	18.91 ± 4.93c	35.87 ± 2.89h	9.67 ± 5.19d	47.65 ± 2.66h

Notes: The data are expressed as mean ± standard deviation. The significance level is set to 0.05 (the same below).

#### 3.2.2 Effects of plant hormone concentration on adventitious shoot regenerations from the callus of *G. rhodantha*

**Figure 2:**
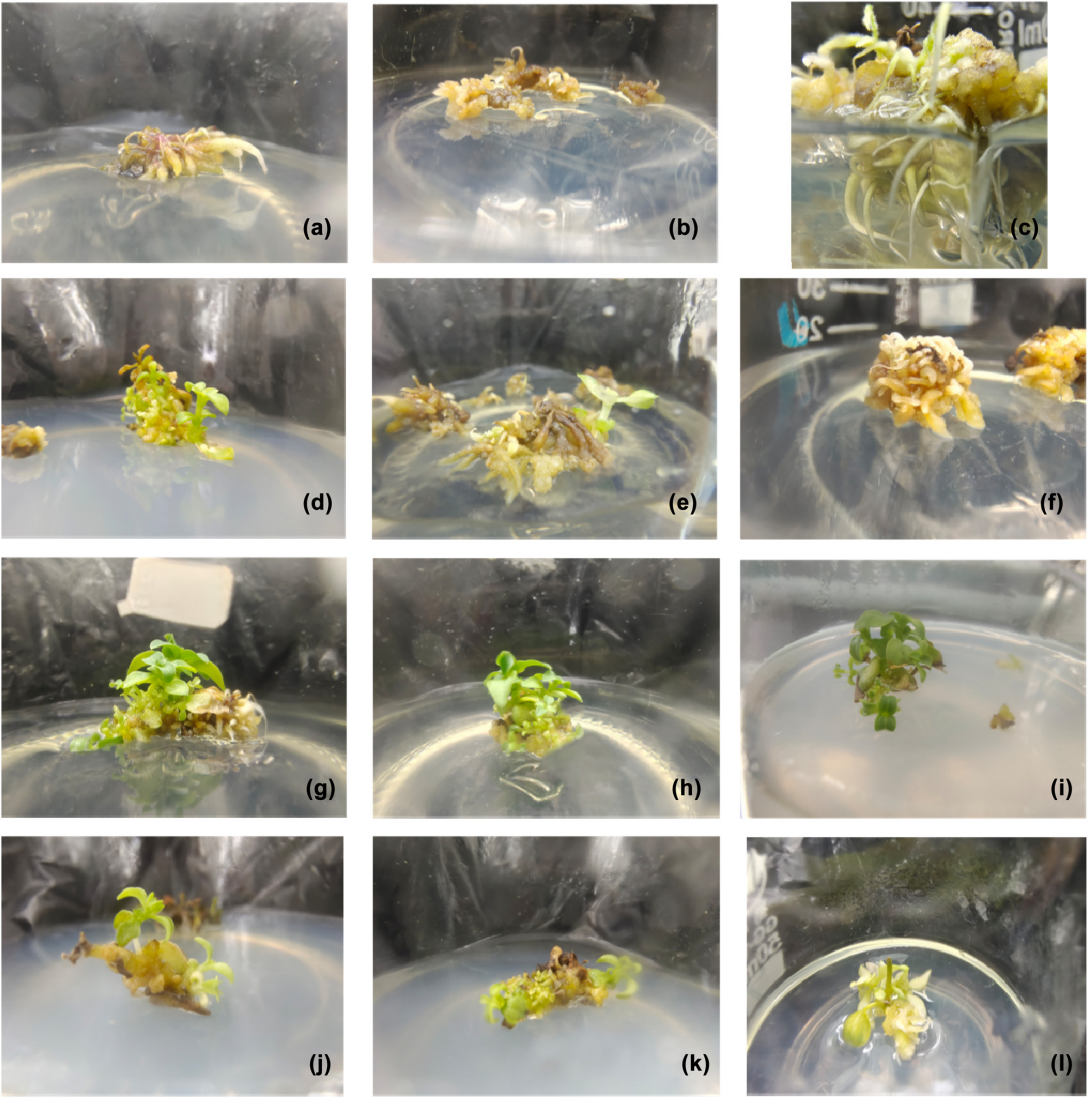
Effects of hormones on adventitious shoot differentiation from the callus of *G. rhodantha.* (a) Adventitious root differentiation from roots was exuberant about 2 weeks later. (b) Dead calluses induced from some roots. (c) Root-induced callus with proliferating root system 40 days later. The calluses were induced from stems after 60 days in culture (when the medium was supplemented with 1.5 mg/L 6-BA and 0.1 mg/L NAA). (d) Calluses were induced from stems after 60 days in culture (when the medium was supplemented with 1.5 mg/L 6-BA and 0.2 mg/L NAA). (e) Calluses were induced from stems after 60 days in culture (when the medium was supplemented with 1.5 mg/L 6-BA and 0.3 mg/L NAA). (f) Calluses were induced from stems after 60 days in culture (when the medium was supplemented with 2.0 mg/L 6-BA and 0.1 mg/L NAA). (g) Calluses were induced from stems after 60 days in culture (when the medium was supplemented with 2.0 mg/L 6-BA and 0.2 mg/L NAA). (h) Calluses were induced from stems after 60 days in culture (when the medium was supplemented with 2.0 mg/L 6-BA and 0.3 mg/L NAA). (i) Calluses were induced from stems after 60 days in culture (when the medium was supplemented with 2.5 mg/L 6-BA and 0.1 mg/L NAA). (g) Calluses were induced from stems after 60 days in culture (when the medium was supplemented with 2.5 mg/L 6-BA and 0.2 mg/L NAA). (k) Calluses were induced from stems after 60 days in culture (when the medium was supplemented with 2.5 mg/L 6-BA and 0.3 mg/L NAA).

The vigorously growing calluses induced from stems and roots were inoculated into the culture media for inducing adventitious shoots. The calluses grew slowly initially, and the texture became more impact. The bud points appeared on the surface of calluses about 1 week later. The adventitious roots induced from the injured roots grew vigorously about 2 weeks later. However, no adventitious shoots were induced (Figure 2a). Some calluses even died over time (Figure 2b). No adventitious shoots were formed after 40 days of culture; the roots thrived and then gradually died (Figure 2c). These observations might be related to the inappropriateness of the hormones used to induce callus differentiation from the roots.

When calluses were induced from stems, the induction rate of adventitious shoots increased with the increase in the concentration (Table 3). However, the induction rate of adventitious shoots decreased as the concentration of 6-BA continued to increase beyond 2 mg/L. No adventitious shoots were induced from stem calluses in the media containing a high concentration of NAA and a low concentration of 6-BA. Besides, the adventitious shoots grew vigorously, with the induction rate of adventitious buds being 0. The adventitious shoots were vitrified when the concentration of 6-BA increased to 2.5 mg/L (Figure 2l). The induction of shoots from stem calluses was the best after treatment with 2.0 mg/L 6-BA and 0.1 mg/L NAA (Figure 2g). The difference was statistically significant compared with that from root and leaf calluses. The shoot regeneration rate reached 86.25%.

**Table 3:** Effects of hormones on adventitious shoots from the callus of *G. rhodantha*

Treatment serial no.	6-BA (mg/L)	NAA (mg/L)	Number of bottles in which explants were inoculated	Induction rate (%)	Growth status	Photos
1	1.5	0.1	20	68.38 ± 4.93c	Vigorous growth, with fewer and slender shoots and dry leaves	D
2	1.5	0.2	20	23.23 ± 1.52f	Weak growth, with root formation but fewer cluster shoots	E
3	1.5	0.3	20	0	No cluster shoots, but with roots formed	F
4	2.0	0.1	20	86.25 ± 4.58a	Vigorous growth, with more, robust shoots induced and roots formed	G
5	2.0	0.2	20	80.36 ± 5.82ab	Vigorous growth, with more, robust shoots induced	H
6	2.0	0.3	20	74.58 ± 4.56b	Vigorous growth, with fewer but robust shoots induced	I
7	2.5	0.1	20	54.34 ± 3.26d	Fewer cluster shoots, occasionally with vitrification	J
8	2.5	0.2	20	46.37 ± 1.15e	Short, slender cluster shoots, with vitrification	K
9	2.5	0.3	20	44.81 ± 4.23e	Short, slender cluster shoots, with severe vitrification	L

Notes: The data are expressed as mean ± standard deviation. The significance level is set to 0.05 (the same below).

#### 3.2.3 Effects of hormone concentration on the propagation of adventitious shoots from *G*. *rhodantha*

**Figure 3:**
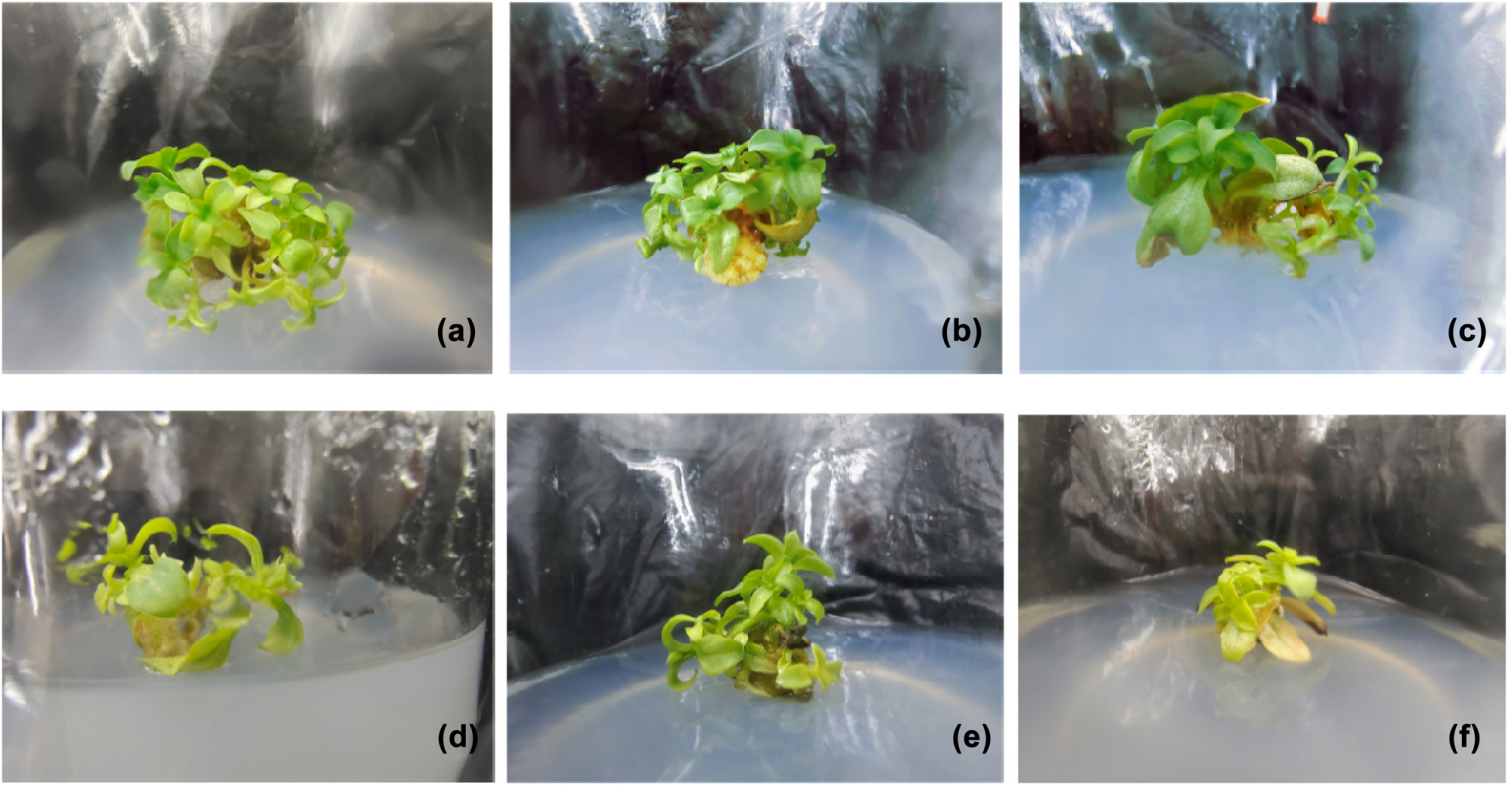
Effects of hormones on the propagation and strengthening of adventitious shoots. Propagation of regenerated shoots when supplemented with: (a) 0.8 mg/L 6-BA and 0.1 mg/L NAA, (b) 0.8 mg/L 6-BA and 0.3 mg/L NAA, (c) 0.8 mg/L 6-BA and 0.5 mg/L NAA, (d) 1.2 mg/L 6-BA and 0.1 mg/L NAA, (e) 1.2 mg/L 6-BA and 0.3 mg/L NAA, and (f) 0.8 mg/L 6-BA and 0.5 mg/L NAA.

Adventitious shoot clusters induced from stems were cut into small pieces of equal sizes along with the corresponding calluses. They were inoculated into the multiplication-inducing culture medium. The propagation index and growth status of adventitious shoots were assessed after 1 month of tissue culture. On this basis, the culture medium most suitable for propagation and shoot strengthening was determined. As shown in Table 4, the cluster shoots were induced from calluses under all concentration combinations of 6-BA and NAA. The growth of adventitious shoots was inhibited by the increasing concentration of 6-BA. The propagation index of adventitious shoots first increased and then decreased as the NAA concentration increased. The propagation index was the lowest when the 6-BA concentration was increased to 1.2 mg/L and the NAA concentration to 0.5 mg/L. Besides, the cluster shoots turned yellow and became severely vitrified (Figure 3f). The propagation index of adventitious shoots was significantly different under the NAA concentration of 0.3 mg/L compared with the concentration of 0.1 and 0.5 mg/L. The propagation index was the maximum when the 6-BA concentration was 0.8 mg/L, corresponding to the vigorous growth of the tissue culture plantlets (Figure 3b). MS supplemented with 0.8 mg/L 6-BA and 0.3 mg/L NAA was the culture medium most suitable for propagation and plantlets strengthening by adventitious shoots induced from *G. rhodantha*, resulting in a propagation index of 8.62.

**Table 4:** Effects of hormones on the propagation of adventitious shoots

Treatment serial no.	6-BA (mg/L)	NAA (mg/L)	Number of inoculated adventitious shoots	Propagation index (K)	Growth status	Photo
1	0.8	0.1	20	7.33 ± 0.15c	Fast propagation, with slender and yellow regenerated shoots	A
2	0.8	0.3	20	8.62 ± 0.15a	Fast propagation, with vigorous growth of regenerated shoots and root formation	B
3	0.8	0.5	20	8.11 ± 0.06b	Fast propagation, with slender regenerated shoots	C
4	1.2	0.1	20	5.21 ± 0.27e	Slow propagation, with slender regenerated shoots and yellow leaves	D
5	1.2	0.3	20	5.98 ± 0.11d	Moderate propagation, with robust growth of regenerated shoots	E
6	1.2	0.5	20	3.51 ± 0.24f	Slow propagation, with yellow and vitrified regenerated shoots	F

#### 3.2.4 Effects of hormone concentration on the rooting of regenerated shoots

**Figure 4:**
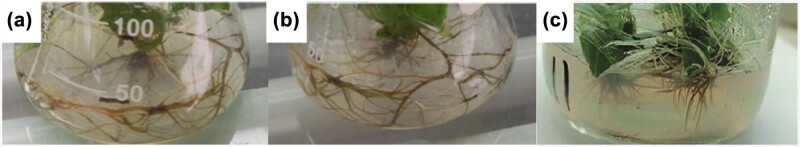
Comparison of rooting with different treatments: (a) root growth on MS medium with 0.1 mg/L IBA, (b) root growth on MS medium with 0.3 mg/L IBA, and (c) root growth on MS medium with 0.5 mg/L IBA.

As shown in Table 5, all three concentrations of IBA could induce the rooting of regenerated shoots, and the rooting rate was consistently 100%. The roots became shorter and more slender under a high IBA concentration, and browning occurred with the increase in the IBA concentration. The average root length per treatment, and the growth and development morphology of roots were compared across the treatments. The MS medium supplemented with 0.3 mg/L IBA was found to be the most suitable for inducing the rooting of regenerated shoots of *G. rhodantha*, which resulted in an average root length of 2.53 cm (Figure 4).

**Table 5:** Effects of IBA concentration on the rooting of adventitious buds of *G. rhodantha*

Treatment serial no.	IBA (mg/L)	Rooting rate (%)	Average root length (cm)	Growth and development morphology
1	0.1	100	2.07 ± 0.11a	Fewer but stockier roots
2	0.3	100	2.53 ± 0.16a	Stockier and developed roots
3	0.5	100	1.67 ± 0.28b	Slender roots

Notes: The data are expressed as mean ± standard deviation. The significance level is set to 0.05 (the same below).

## 4 Discussion and conclusion

The presence of endophytes in *Gentiana* plants [15] usually results in a high explant contamination rate during tissue culture [16–19]. We determined the optimal disinfection method for the stem and root segments, which involved disinfection with 75% ethanol for 50 s, followed by 4% NaClO for 10 min. For leaves, the optimal disinfection method consisted of disinfection with 75% ethanol for 50 s, followed by 4% NaClO for 8 min. The stems, roots, and leaves were then used as sterile explants after the disinfection procedure.

The present study used leaves, stems, and roots as explants. In the explants disinfection experiment, we found that the leaves, young roots, and young stems of *G. rhodantha* were more tender and susceptible to browning and death. The stem and roots had lower browning rates and higher survival rates than the leaves. This finding agreed with the results obtained by Huo [18] and Guo et al. [20]. Wang [19] found that the callus induction rate of young leaves in *Conyza blinii* H. Lev was higher than that of old leaves, although the differentiation rate remained low. This result agreed well with our finding of a lower induction rate and higher browning rate of the leaf calluses of *G. rhodantha*. This was probably because the *Gentiana* plants are rich in flavones, leading to a higher possibility of browning.

The type and concentration of exogenous hormones are closely related to the number and growth status of adventitious shoots induced from plants. The ratio of cytokinin to auxin is a key factor determining callus differentiation. In the present study, the differentiation rate of adventitious shoots from stems first increased and then decreased as the 6-BA concentration increased. As the 6-BA concentration increased to 2.5 mg/L, the adventitious shoots were shorter, slender, and severely vitrified. That is, an excessively high concentration of 6-BA had an inhibitory effect on adventitious shoot differentiation. No adventitious shoots were differentiated, and the root system grew exuberantly under the combination of low-concentration 6-BA and high-concentration NAA. The root-induced callus treated with the conventional hormone combination of 6-BA and NAA had no differentiation of adventitious shoots. No adventitious shoots germinated, and the roots grew vigorously under the joint action of a low concentration of 6-BA and a high concentration of NAA. The following conclusions were drawn from these observations: (1) *G. rhodantha* is a perennial herb with developed roots and remarkable rooting ability; therefore, many adventitious roots were induced from the root callus, but no adventitious buds were germinated [21]. (2) Root formation using high concentration of NAA could be due to the known effect of auxin, i.e., high auxin concentrations promote root formation. Thus, only a proper ratio of auxin and cytokinin can induce root and shoot regeneration.

During secondary culture, an increase in the propagation index of adventitious shoots depended on the increase in cytokinin concentration. However, as the cytokinin concentration continued to increase, the adventitious shoots showed poor growth and became shorter and slender, making the root culture difficult. Even if the roots were formed, the transplanted tissue culture plantlets were slender and had low stress resistance. Such plantlets might not survive and require plantlets-strengthening culture. According to our experiment, the MS supplemented with 0.8 mg/L 6-BA and 0.3 mg/L NAA culture medium was more suitable for propagation by adventitious shoots and plantlets strengthening, with the maximum propagation index being 8.6.

Our experiments showed that *G. rhodantha* had a good rooting ability. The rooting rate was consistently 100%, regardless of the root-inducing medium used. Besides, rooting was also observed in the adventitious bud and propagation induction experiments. MS supplemented with 0.3 mg/L IBA was the optimal culture medium for inducing the rooting of adventitious buds from *G. rhodantha*, with a rooting rate of 100%. This was probably because *Gentiana* plants were perennial plants with strong rooting ability.

The growth hormones, explant type, and type of endogenous hormones in plants have a significant influence on the regeneration capacity of *G. rhodantha*. Our results showed that the adventitious shoots could be differentiated from the callus induced from stem explants, and rooting was successfully induced. The rapid propagation system of *G. rhodantha* was built on this basis, providing technical references for rapid growth of plantlets and large-scale cultivation.
